# HAE international home therapy consensus document

**DOI:** 10.1186/1710-1492-6-22

**Published:** 2010-07-28

**Authors:** Hilary J Longhurst, Henriette Farkas, Timothy Craig, Emel Aygören-Pürsün, Claire Bethune, Janne Bjorkander, Konrad Bork, Laurence Bouillet, Henrik Boysen, Anette Bygum, Teresa Caballero, Marco Cicardi, John Dempster, Mark Gompels, Jimmy Gooi, Sofia Grigoriadou, Ursula Huffer, Wolfhart Kreuz, Marcel M Levi, Janet Long, Inmaculada Martinez-Saguer, Michel Raguet, Avner Reshef, Tom Bowen, Bruce Zuraw

**Affiliations:** 1 Department of Immunology, Barts and the London NHS Trust, London, UK; 2 3rd Department of Internal Medicine, Faculty of Medicine, Semmelweis University, Budapest, Hungary; 3 Departments of Medicine and Pediatrics, Penn State University, Hershey, Pennsylvania, USA; 4 Johann Wolfgang Goethe University, Frankfurt/Main, Germany; 5 Department of Immunology, Plymouth Hospitals NHS Trust, UK; 6 Dept of Internal Medicin, Ryhov County Hospital, SE-55185 Jönköping, Sweden; 7 Department of Dermatology, University Hospital of the Johannes Gutenberg-University of Mainz, Mainz, Germany; 8 Department of Medicine, CHU de Grenoble, Grenoble, France; 9 Executive Director, HAE International, Denmark; 10 Department of Dermatology and Allergy Centre, Odense University Hospital, Denmark; 11 Hospital La Paz Health Research Institute, Madrid, Spain; 12 Department of Internal Medicine, Universita degli Studi di Milano, Ospedale L. Sacco, Milan, Italy; 13 Department of Immunology, Barts and the London NHS Trust, London, UK; 14 Department of Immunology, Southmead Hospital, Bristol, UK; 15 Department of Immunology, St James' Hospital, Leeds, UK; 16 Department of Immunology, Barts and the London NHS Trust, London, UK; 17 HAE association, Germany; 18 Johann Wolfgang Goethe University, Frankfurt/Main, Germany; 19 Academic Medical Center, University of Amsterdam, Amsterdam, Netherlands; 20 US HAEA Executive Vice President; US HAEA Patient Registry, USA; 21 Johann Wolfgang Goethe University, Frankfurt/Main, Germany; 22 HAE Association, France; 23 Tel Hashomer, and Sackler Faculty of Medicine, Tel Aviv University, Ramat Aviv, Israel; 24 Departments of Medicine and Paediatrics, University of Calgary, Calgary, Alberta, Canada; 25 University of California, San Diego, San Diego, California, USA

## Abstract

Hereditary angioedema (C1 inhibitor deficiency, HAE) is associated with intermittent swellings which are disabling and may be fatal. Effective treatments are available and these are most useful when given early in the course of the swelling. The requirement to attend a medical facility for parenteral treatment results in delays. Home therapy offers the possibility of earlier treatment and better symptom control, enabling patients to live more healthy, productive lives. This paper examines the evidence for patient-controlled home treatment of acute attacks ('self or assisted administration') and suggests a framework for patients and physicians interested in participating in home or self-administration programmes. It represents the opinion of the authors who have a wide range of expert experience in the management of HAE.

## Introduction

Hereditary angioedema (HAE) is an autosomal dominant condition caused by a partial deficiency of C1 inhibitor (C1INH). C1INH controls a variety of local inflammatory pathways. Insufficient regulation of the classical complement pathway causes consumption of the complement component C4, resulting in typical diagnostic abnormalities. Insufficient inhibition of kallikrein results in overproduction of bradykinin, and episodic swelling from excess local bradykinin[1]. Swellings are typically of slow onset over several hours and last 1-5 days, although some patients may also experience rapid onset swellings[2]. Almost any part of the body may be affected, although the subcutaneous and submucosal structures of limbs, genitals, face, mouth and bowel are the usual sites of swelling. Swellings are referred to as acute attacks and are interspersed by asymptomatic periods of days, weeks or months[3-5].

Attacks are associated with reversible disability. Abdominal attacks are associated with the extreme pain of bowel obstruction or visceral swelling, and with vomiting or diarrhoea[3,6]. Patients are typically unable to undertake their daily activities of living for one or more days, and may be less productive for a few days subsequent to attack because of residual angioedema and fatigue. Peripheral swellings may prevent wearing of shoes, operation of machinery or may cause disfigurement, thus interfering with work or other activities[7].

Laryngeal swellings comprise a small minority (around 2%) of attacks but may cause death from airway obstruction[2]. Although for most patients laryngeal attacks are infrequent and the case fatality of each attack is low, there is a significant lifetime mortality[3,8]. Fear of laryngeal attacks and the need for access to lifesaving, specialist emergency treatment confers considerable restrictions on patients and their families. In particular, travel for work or pleasure is often curtailed. Attacks are more likely to occur at times of emotional stress, and therefore are more likely to occur during examinations or busy periods at work. Thus, those affected by HAE and their families are subject to employment and educational disadvantage[9-11].

## Treatment options

The frequency and severity of attacks is reduced by oral prophylaxis with attenuated androgens or tranexamic acid, or by regular intravenous infusion of C1INH concentrate[12-14]. However, prophylaxis does not completely abolish attacks[14]. Moreover, many patients cannot benefit from oral prophylaxis because of contraindications, side effects, or lack of efficacy[13,15,16]. Immediate access to effective acute treatment is therefore required for all patients.

Replacement therapy with plasma-derived C1INH (pdC1INH) is effective and has been used successfully for over 25 years to attenuate or prevent attacks at all sites [17-19]. More recently, icatibant, a bradykinin receptor inhibitor, and ecallantide, a kallikrein inhibitor, have been shown to be effective at treating attacks[20,21]. Icatibant was licensed for acute angioedema attacks in patients with C1-inhibitor deficiency in the European Union and several other countries in 2008. Ecallantide was licensed for acute attacks in the USA in 2009. After treatment with either icatibant, ecallantide or C1INH, onset of relief can be expected within 30 to 60 minutes, with full resolution taking a few hours to longer than 24 hours in established attacks[14,17,20-23]. Observational studies demonstrate faster relief and reduced attack severity when C1INH is given early [6,24]. Similarly, icatibant or ecallantide may be more effective when given early, although this is yet to be demonstrated in clinical studies.

The requirement to travel to a medical facility for acute treatment implies at least half a day away from home, school, or work. In the emergency room setting, patients with HAE attacks may not be prioritised over competing emergencies such as patients with heart attacks, stroke or severe injury. This leads to further delay, with increased risk of treatment failure and longer recovery times. Health care staff, unfamiliar with HAE, are reluctant to treat early, when signs are mild or absent, contributing further to the delay and disability. Owing to the reversibility of most attacks, patients themselves may choose to stay at home with symptomatic treatment rather than struggle with access to therapy in the emergency room. This increases absenteeism from work, school, or home responsibilities. For laryngeal attacks, such delay may be fatal. Even where acute treatment is available, it is estimated that only 5-25% of attacks receive definitive treatment (Cicardi, personal communication). Home infusion programmes have been established in centres with an interest in HAE, and have the potential to overcome many of the difficulties associated with health care facility-based treatment[25-28].

Experience shows that access to self or assisted infusion with C1INH reduces severity and duration of attacks, improves HAE-related quality of life, reduces time off work, education or domestic duties, is safe, and is very popular with participants[23-30]. Self infusion is endorsed by the UK, Danish and Canadian-Hungarian consensuses[25,31-34]. Neither UK nor the Canadian Consensus documents place restrictions on entry to the programme, other than to note that the diagnosis of HAE should be proven, prophylactic therapy optimised (UK document) and that an 'infusion partner' (UK) or 'instruction and support' (Canadian- Hungarian) should be available[27,31-34]. The Danish home therapy programme considers patients who have more than one moderate or severe attack every 4 weeks, although in practice, these restrictions are relaxed where necessary[25,27].

The C1 inhibitor deficiency workshop was first held in 1999 in Vizegrád, Hungary and has been a biennial Hungarian event since then. It brings together patients, physicians, nurses, basic and clinical scientists, blood product suppliers and representatives of the pharmaceutical industry with a common interest in HAE and its management. Similarly, the Canadian Hereditary Angioedema Network (CHAEN)/Réseau d'angioedème héréditaire du Canada (RAHC) has organised conferences in 2003, 2006 and 2010, with the aim of developing and updating consensus recommendations for HAE management http://www.haecanada.com. These workshops are attended by experts from all over the world and represent an unprecedented opportunity to pool experience and to share information, with the aim of improving the physical, psychological and economic wellbeing of those affected by HAE. This article summarises consensus opinion, resulting from debates at the Hungarian C1 Inhibitor 2009 Workshop and the Canadian Hereditary Angioedema Network (CHAEN)/Réseau d'angioedème héréditaire du Canada (RAHC) consensus meeting held in Toronto in May 2010 (Appendix 1). **Home therapy/self-administration in this article, refers to treatment given in a non-health care setting; given by the patient, a trained relative or friend, outside of a healthcare facility**.

## Methods

Participants at the 6^th ^C1 Inhibitor Deficiency Workshop, held in Budapest in May 2009, were invited to a moderated discussion with the aim of forming a consensus approach to home therapy for patients with HAE. Published literature was found using a PubMed search, with the terms "C1 inhibitor" or "hereditary angioedema" and "home therapy" or "self-infusion". Additional data were found from references in the papers found in the search and from abstracts known to the authors, not listed in PubMed. These data were summarised by HL and presented as a basis for further discussion. The discussion was chaired by a physician/immunologist (HL) and moderated by a representative of the US patients' association (JL), adult and paediatric physicians, and physician scientists (TC, WK, BZ). The consensus document summarising discussions was drafted by HL and the facilitators (TC, WK, BZ). This draft was used as a basis for debate and modification at the Canadian Toronto May 2010 Consensus Meeting. To date, most self-administration treatment has been limited to plasma-derived C1INH (pdC1INH) replacement but is expected to involve other agents as they become available (icatibant, recombinant C1INH, other therapies as they become licensed and available).

## Recommendations

### 1. Inclusions/exclusions

**Recommendation: **Every patient with HAE should be considered for home therapy and self-administration training, once the diagnosis of C1INH deficiency (hereditary or acquired angioedema) is confirmed.

Prophylaxis should be optimised, but home therapy need not be delayed since training can take place alongside other treatments. Attacks occur despite prophylaxis in most patients. Requirement to attend a medical facility inevitably results in delayed treatment, and many attacks are not treated at all. This contributes to the social and economic disadvantage associated with HAE. For this reason, eligibility for home therapy should be inclusive. It is strongly recommended that patients train with an 'home therapy partner' (a family member or friend who can provide support, advice and may additionally be trained to perform the therapy administration).

#### Special situations

(i) Extremes of age;

**1. Children**: C1INH home therapy is recommended for children with frequent or disruptive attacks, where a responsible adult is available and willing to undertake training. Experience with haemophilia suggests that it is beneficial for children to be encouraged to take an active part in their treatment with a view to independent administration in their early teens [35].

**Comment**: Prepubertal children typically experience fewer attacks than adolescents and adults. However, some have frequent attacks which disrupt education and family life[36,37]. Prophylaxis with attenuated androgens is not recommended, and tranexamic acid may be of limited benefit[13]. pdC1INH is effective for treatment of children and home therapy has been used successfully in this age group (W Kreutz; personal communication). Icatibant has not been tested in children under 18 years and is not yet recommended for this age group.

**2. Elderly**: Advanced age is not a contraindication to home therapy, providing patient and home therapy partner can function safely and effectively.

**(ii) Pregnancy and breastfeeding**: pdC1INH home therapy appears safe and effective and is recommended for pregnant and lactating women[28,32,38].

**Comment: **Attack frequency increases in the first trimester of pregnancy for approximately 38% [36] of women. Prophylaxis with attenuated androgens or tranexamic acid is not recommended in pregnancy. Home or self-administration should be considered for control of symptoms for pregnant and lactating women. Women with HAE contemplating pregnancy should consider home self-administration training.

**(iii) Lack of home therapy partner**: While presence of a trained 'home therapy partner' is highly desirable, lack of a partner does not exclude the possibility of self-administration, if a risk-benefit assessment is favourable

**Comment: **Those without an infusion partner may include students and patients undertaking travel for work purposes. Patients living alone may experience particular difficulty in travelling to hospital, and therefore may be more likely to present late or to leave severe attacks untreated if unable to self-administer. These groups are likely to gain the greatest benefit from the ability to prevent and control attacks. We recommend that extra care is taken with arrangements for access to medical back-up where an infusion partner is not available. Subcutaneous agents such as icatibant could be considered as an alternatives to pdC1INH for therapy because of ease of administration. However, more experience is needed (see below). Ecallantide is not currently recommended for selfadministration because of the risk of anaphylactoid reactions.

### 2. Attack Treatment; infusion timing and regimen

Treatment is recommended as soon as the patient identifies symptoms, which, if untreated, are likely to develop into a moderate or severe attack.

Comment:

Attack treatment with pdC1INH, rhC1INH, icatibant, or ecallantide is likely to be most effective when given early in the course of the attack [22]. Individualised 'on demand treatment' during the initial or prodromal stages of an attack enables patients to achieve almost complete freedom from symptoms [24]. The dose of pdC1INH treatment may be individually adjusted depending on response. For airway events, the dose should be 20 units/kg rounded up to the next highest vial[18]. For treatment of other attacks, the best dosage and timing of administration may differ from that reported in randomised controlled studies which necessarily evaluates fixed regimens in established attacks[18]. Individual requirements may be evaluated by the patient, with guidance from the physician. Many patients have found doses lower than the licensed dose of 20 units/kg pdC1INH effective, with doses of 500-1000 units sufficing for many attacks[22,37,39,40]. A dose-finding trial for home therapy with pdC1INH is needed.

### 3. Prophylaxis

Short term prophylaxis with pdC1INH is recommended for high risk events. In patients with frequent attacks this may include emotionally stressful events such as exams, interviews, busy work periods and major family events, as well as cover for medical and dental procedures.

Long-term pdC1INH prophylaxis may be required, in some cases, to control very frequent attacks.

**Comment: **pdC1INH is effective in short and long-term prophylaxis. Controversy exists as to the relative advantages of long-term prophylaxis compared with treatment of early symptoms. PdC1INH (Cinryze™) is licensed in the USA for long-term prophylaxis (1000 units twice weekly). Long-term prophylaxis reduces attack frequency and severity, and improves quality of life [14]. However, breakthrough attacks are frequent and the total usage of pdC1INH is often increased[14,26]. Although control may be improved by adjustment of dose and frequency of treatments, many, but not all, experts prefer early symptomatic treatment.

Icatibant, ecallantide and rhC1INH have short half-lives and are therefore not recommended for prophylaxis at this time[20,21,41].

### 4. Route of administration

**Recommendation**: Most experts recommend venepuncture with a small (e.g. 28G) butterfly needle infusion set on each occasion that treatment with C1INH is required.

**Comment: **Self-administration is associated with a low incidence of cannulation failure and may preserve veins more effectively than hospital-based care[26]. Indwelling central line devices may be considered in exceptional cases where venous access in a timely manner would otherwise not be possible. However, the requirement for treatment is lifelong and indwelling devices have a finite life. Complications associated with indwelling venous ports, particularly infection and occlusion, may be serious and are also likely to increase the frequency of attacks. For these reasons, venous ports should be avoided where possible. Icatibant given subcutaneously may be considered where venous access is difficult.

### 5. Site of attack

**Recommendation: **Patients should be encouraged to treat any attack at any site interfering with activities of daily living or which is likely to be associated with further disabling attacks.

**(i) Laryngeal/upper airway attacks**: Attacks affecting head and neck should always be treated even if symptoms are mild because of the potential for rapid progression to laryngeal obstruction[2]. Patients are strongly advised to seek emergency medical assistance for all intraoral attacks and should home-administer treatment while awaiting transfer to hospital.

**(ii) Cutaneous attacks**: Treatment with pdC1INH, rhC1INH, icatibant or ecallantide is effective. Mild attacks may not require treatment or may respond to oral tranexamic acid[13]. Definitive treatment is recommended for attacks that have potential to interfere with daily activities.

**(iii) Abdominal attacks: **Treatment is likely to reduce HAE-related disability and socioeconomic disadvantage.

**Comment: **With the exception of attacks affecting the head and neck, which may progress to laryngeal obstruction[2] symptoms are self limiting after 1-5 days and patients may choose to leave these untreated or to use symptomatic treatment.

### 6. Counselling/consent

**Responsibilities of the doctor**: We recommend that:

(i) The physician is responsible for carrying out an assessment of the risks and benefits of home-administration. They should ensure that the patient and treatment partner are able to provide fully informed consent, in particular with respect to:

1. Blood product origin of pdC1INH

2. Appropriate treatment of attacks

3. Management of HAE-related emergencies

4. Management of treatment-associated side effects

(ii) The physician should ensure that a treatment plan for 24 hour local emergency assistance and specialist advice are available and that the patient and local hospital have written information.

(iii) The patient should be issued with appropriate emergency treatment (at least one treatment dose pdC1INH), as this is unlikely to be immediately available in every medical facility.

(iv) The physician is responsible for ensuring that the patient and partner are competent with the medical and technical aspects of home administration. On-line resources provide helpful support [for example: http://www.haecanada.com]. These should not be a substitute for direct access to advice from the specialist centre.

In practice, training may be delegated to a trained specialist nurse.

**Responsibilities of the patient/treatment partner**: We recommend that:

(i) The patient, with the support of the administration partner, should be prepared to take responsibility for the decision to treat, the technical aspects of safe use of pdC1INH or other acute treatment and the safe disposal of used equipment. Patients should keep an accurate note of treatments and dates, including the lot numbers of products used.

(ii) Patients should undertake to attend regular follow up and refresher training, and to seek prompt assistance from their emergency medical facility or from their physician or specialist nurse in the event of any query or difficulty.

(iii) Patients should be aware of, and should accept, the partial transfer from the physician of responsibility for their medical care, and the wider responsibility to the HAE community of ensuring that home administration is practised safely and effectively.

The patient should retain the option of returning to hospital/clinic-based acute care at any time, or of leaving the home-administration programme.

Written consent is advised.

### 7. Training programme

**Recommendation: **The training programme, which may be led by an appropriately trained and experienced specialist nurse or physician, should be conducted over several sessions to ensure that patient and treatment partner have had sufficient practice to be familiar and confident with technical and medical aspects of self-administration.

The patient should have received the proposed home treatment for an attack on at least one occasion prior to home therapy. Ideally, the patient/partner should treat an attack under medical supervision, prior to home therapy. However, in practice this may not always be possible.

When training is complete, there should be an assessment to ensure that the patient and partner's knowledge and technical ability are sufficient.

Refresher training should be planned at regular intervals, usually at least every 12 months.

Training should include:

Appropriate use of treatment product(s) (pdC1INH, rhC1INH, icatibant) Management of emergencies, including when to seek professional help.

Supply & storage of treatment products Handwashing and aseptic technique Preparation of equipment Product checking (dose/expiry date) Reconstitution of products if needed Intravenous access Administration of medication, including infusion rate Disposal of equipment Record keeping (batch number, attack record)

### 8. Other HAE-like syndromes

#### (i) Acquired C1 inhibitor deficiency

Patients with acquired C1INH deficiency may also benefit from home therapy. In some, but not all cases, higher doses of C1INH may be required[26,42]. Icatibant is likely to be an attractive option in this case [43].

#### (ii) HAE type 3

HAE type 3, although similar to other hereditary angioedemas, is not associated with C1INH deficiency [44-46]. This document does not consider HAE3, although if effective acute treatments are identified, the same principles will apply.

### 9. Emerging therapies

PdC1INH has the widest availability and licensure. Icatibant and ecallantide have variable licensure and rhC1INH is applying for licensure. Availability of products will vary by jurisdiction.

#### Icatibant

**Recommendation: **Icatibant, where available, may be considered as an alternative to C1INH for home therapy.

**Comment: **Icatibant has the advantage of subcutaneous administration and, unlike C1INH, is supplied in a prefilled syringe. Approximately 10% will need a second dose of icatibant[20], and patients should hold a backup dose.

By reducing the need for technical expertise, icatibant has the potential to revolutionise the management of HAE. However, it is not yet licensed for self-administration and experience is very limited. There is no experience of icatibant in children nor pregnant or lactating women and for this reason, icatibant cannot currently be recommended for these groups. Further information from currently ongoing self-administration trial and registry data will be vital in strengthening this recommendation.

**Ecallantide**: As with icatibant, ecallantide is administered by the subcutaneous route. Anaphylactic reactions have been reported[47] and for this reason, the FDA has mandated a registration program that requires an informed consent that treatment with ecallantide only be performed in the office of a physician experienced and equipped to treat anaphylaxis. In light of this self-treatment at home is not yet possible for ecallantide. At the present time, due to lack of data, ecallantide is not recommended in pregnant, nor lactating women, or children under the age of 16 years.

**Recombinant C1 inhibitor**: Rhucin, a recombinant C1INH, is currently in development. It is likely to be an alternative to pdC1INH for the treatment of acute attacks. It has a shorter half-life than pdC1INH, therefore its role in prophylaxis is uncertain[41].

## Conclusion

Every patient with HAE should have the immediate means to control an acute attack quickly and effectively, in order to minimise impact on physical, social and economic wellbeing to themselves and their family. Similar to the haemophilia home care model that has worked so well for many years[35], the option of home and self-administration offers the prospect of achieving the aim of 'each C1 inhibitor deficient (HAE) patient to be able to manage his/her symptoms proactively in such a way that they maintain personal safety and minimal disruption in living a healthy and productive life'[32].

## Appendix 1. Recommendations: summary

### Inclusions

Home therapy training should be inclusive.

Extremes of age or lack of infusion partner are not necessarily contraindications. Patients with HAE, with acquired angioedema (acquired C1 inhibitor deficiency), and, where treatment is available, HAE3, should be included.

Prophylaxis, if required, should be optimised while home therapy training is ongoing.

### Attack Treatment

Attacks should be treated as early as possible.

Dose of C1 inhibitor should be individualised.

Prophylactic pdC1 inhibitor regimens may occasionally be necessary.

### Site of attack

Attacks at all sites may be self-treated at home. In the case of laryngeal oedema, urgent transfer to hospital is recommended after treatment.

### Counselling/consent

Physician and patient should take joint and individual responsibility for the safe and appropriate use of home therapy.

### Training programme

Should take part in a centre experienced in HAE management.

Should offer ongoing support and refresher training.

### Emerging therapies

pd C1 inhibitor home therapy programmes are well established.

rC1 inhibitor is likely to offer an alternative for acute treatment when licensed.

Icatibant offers great potential for expanding access to treatment because of its ease of administration.

Ecallantide is not recommended for home therapy

## Competing interests

Many of the authors have either entered consultancy with or have been involved in educational programs and their organization, had direct funding from, have been speakers for, or have had consultation agreements with CSL Behring, Dyax, Jerini, Pharming, ViroPharma, Shire. The HAE International Home Therapy Consensus Document was arrived at following debate during the Hungarian C1 Inhibitor 2009 Workshop, held in Budapest May 22/24, 2009 and the Canadian Hereditary Angioedema Network (CHAEN)/Réseau Canadien d'angioédème héréditaire (RCAH) second meeting held May 15^th^/16^th^, 2010, Toronto, Canada. CHAEN was cosponsored by CHAEN/RCAH, the Canadian Society of Allergy and Clinical Immunology, and the University of Calgary and was funded through an unrestricted educational grant from CSL Behring. Publication of this manuscript is sponsored by University of Calgary.

## Authors' contributions

HL chaired home therapy debates and facilitated consensus at the Budapest workshop and Toronto CHAEN meetings. TC, JL, WK, BZ acted as faciliators/expert panel at the debate at the Budapest workshop. HL prepared the manuscript. All authors have read, revised and approved the manuscript and have participated in one or more of the debates, or in the subsequent discussions.
